# Longitudinal Changes in Caregiver Psychological Well-Being and Infant Temperament Following Preterm Birth: From Enrollment to 12 Months Corrected Age

**DOI:** 10.3390/children13101295

**Published:** 2026-09-24

**Authors:** Dohyung Lee, Nam Hyun Lee, Sumin Kim, Eun Jae Ko

**Affiliations:** 1 University of Ulsan College of Medicine, Seoul 05505, Republic of Korea; st00303@amc.seoul.kr; 2 Department of Rehabilitation Medicine, Asan Medical Center, Seoul 05505, Republic of Korea; rptlee@amc.seoul.kr; 3 Department of Rehabilitation Medicine, Asan Institute for Life Sciences, Foundation for Industry Cooperation University of Ulsan, Seoul 05505, Republic of Korea; suemin5079@amc.seoul.kr; 4 Department of Rehabilitation Medicine, Asan Medical Center, University of Ulsan College of Medicine, 88, Olympic-Ro 43-Gil, Songpa-Gu, Seoul 05505, Republic of Korea

**Keywords:** preterm infants, caregivers, parenting stress, depression, infant temperament, longitudinal studies

## Abstract

**Highlights:**

**What are the main findings?**

- In this 12-month longitudinal cohort of preterm infant–caregiver dyads, caregiver depressive symptoms and parent-domain parenting stress decreased over time.
- Infant Surgency/Extraversion and Negative Affectivity increased over time.

**What are the implications of the main findings?**

- Caregiver psychological well-being and infant temperament change dynamically during the first corrected year after preterm birth.
- Longitudinal, family-centered follow-up may help identify dyads who could benefit from early supportive interventions.

**Abstract:**

**Background:** Advances in neonatal care have substantially improved the survival of preterm infants; however, longitudinal changes in caregiver psychological well-being and infant temperament during follow-up remain incompletely understood. This study aimed to characterize changes in caregiver psychological measures and infant temperament from enrollment through 12 months corrected age (CA) and to explore their clinical and socioeconomic correlates. **Methods:** This prospective longitudinal cohort study included 35 preterm infant–parent dyads assessed at enrollment (mean CA, 2.3 ± 1.3 months), 6 months CA, and 12 months CA. The same parent completed measures of depressive symptoms (Korean Beck Depression Inventory-II [K-BDI-II]), parenting stress (Korean Parenting Stress Index, Fourth Edition [K-PSI-4]), general self-efficacy, and infant temperament (Infant Behavior Questionnaire–Revised [IBQ-R]) across available assessments. Developmental outcomes were assessed using the Bayley Scales of Infant Development, Second Edition (BSID-II) at 12 months CA. Longitudinal changes were analyzed using linear mixed-effects models with false discovery rate (FDR) correction across the eight domain-level questionnaire outcomes comprising the main longitudinal analysis family; additional analyses were exploratory. **Results:** K-BDI-II depressive symptom scores (FDR q = 0.022) and K-PSI-4 Parent-domain stress (q = 0.011) decreased over time, whereas Total Stress, Child-domain stress, and general self-efficacy did not change significantly. Infant Surgency/Extraversion (q < 0.008) and Negative Affectivity (q = 0.008) increased, whereas Orienting/Regulation did not change significantly. No associations between clinical or socioeconomic characteristics and study outcomes remained significant after FDR correction. At 12 months CA, K-PSI-4 Total Stress was positively correlated with parent-reported infant Negative Affectivity (r = 0.47, q = 0.039). **Conclusions:** Caregiver psychological measures and infant temperament showed distinct longitudinal changes from enrollment through 12 months CA following preterm birth. Repeated assessment of caregiver psychological well-being may complement developmental follow-up of preterm infants. These within-cohort findings, particularly the exploratory associations, require confirmation in larger and more diverse longitudinal cohorts.

## 1. Introduction

Advances in neonatal care have substantially improved the survival of preterm infants over recent decades. Nevertheless, preterm birth remains associated with an increased risk of long-term neurodevelopmental, behavioral, and psychosocial difficulties that may extend into childhood and adulthood [1,2,3]. Although follow-up programs have traditionally focused on physical growth and development, increasing attention has been directed toward broader developmental outcomes, including behavioral regulation and family well-being.

The consequences of preterm birth extend beyond the infant to the entire family. Parents of preterm infants often experience considerable psychological distress related to prolonged hospitalization, medical uncertainty, and concerns about their child’s future development [4]. Elevated depressive symptoms and parenting stress may influence parent–infant interactions, which are increasingly recognized as relevant to early development. A family-level framework for considering these processes is provided by the Family Stress Model, which proposes that contextual stressors may influence child adjustment in part through caregiver psychological distress and family or parenting processes [5]. Although this framework was developed primarily in the context of economic hardship, it provides a useful conceptual lens for considering how medical, caregiving, and socioeconomic stressors following preterm birth may interact with caregiver well-being and the family environment over time. Longitudinal assessment of caregiver psychological well-being and infant behavioral characteristics may therefore provide a broader understanding of changes occurring within the family context during follow-up after preterm birth.

Despite growing recognition of family-centered care, longitudinal evidence regarding caregiver psychological well-being after preterm birth remains limited. Previous studies have generally examined individual aspects of caregiver mental health or infant developmental outcomes separately, whereas relatively few have simultaneously evaluated caregiver depressive symptoms, parenting stress, general self-efficacy, infant temperament, and developmental outcomes within the same longitudinal cohort. Furthermore, little is known about how caregiver psychological well-being and infant temperament change concurrently during infancy or how clinical and socioeconomic characteristics are associated with these outcomes.

Accordingly, this prospective longitudinal study followed preterm infants and their parent respondents from enrollment through 12 months corrected age (CA). Specifically, we aimed to (1) characterize longitudinal changes in caregiver psychological well-being and infant temperament from enrollment through 12 months CA; (2) explore clinical and socioeconomic characteristics associated with caregiver psychological well-being, infant temperament, and developmental outcomes; and (3) evaluate concurrent and longitudinal relationships between caregiver psychological measures and infant temperament or developmental outcomes.

## 2. Materials and Methods

### 2.1. Study Design and Participants

This prospective longitudinal observational cohort study was conducted at the Department of Pediatric Rehabilitation Medicine, Asan Medical Center, Seoul, Republic of Korea. Recruitment was conducted between 24 April 2023 and 5 July 2025. The study was conducted in accordance with the Declaration of Helsinki, and the protocol was approved by the Institutional Review Board of Asan Medical Center (No. 2023-0497) and registered with the Clinical Research Information Service of the Korea Disease Control and Prevention Agency (KCT0011954).

The source population consisted of preterm infants receiving follow-up care at the pediatric rehabilitation outpatient clinic. Potentially eligible infant–caregiver dyads were approached consecutively during outpatient visits. The inclusion criteria were: (1) preterm birth before 37 completed weeks of gestation; (2) attendance at the pediatric rehabilitation outpatient clinic before 6 months CA; and (3) availability of a primary caregiver who could participate in the study. Dyads were excluded if the primary caregiver did not provide written informed consent for study participation.

A total of 105 eligible infant–caregiver dyads provided initial consent and were enrolled. Sixteen caregivers subsequently withdrew before baseline assessment, leaving 89 dyads who completed the baseline assessment and entered longitudinal follow-up. During follow-up, 54 dyads did not complete the 12-month follow-up: 18 were lost to contact and 36 withdrew consent. Ultimately, 35 dyads were retained through the 12-month follow-up and comprised the longitudinal analytic cohort. Participant recruitment, assessment schedule, and measure availability are summarized in Figure 1.

The questionnaire respondent was distinguished from the infant’s primary day-to-day caregiver, defined as the person primarily responsible for the infant’s daily care. All questionnaire respondents in the analytic cohort were parents (33 mothers and 2 fathers). For each infant–parent dyad, the same parent completed all available K-BDI-II, K-PSI-4, general self-efficacy, and IBQ-R assessments across the three study visits. Grandparents or babysitters identified as primary day-to-day caregivers did not serve as questionnaire respondents. One pair of twins was included; the mother served as the questionnaire respondent for one twin and the father for the other, such that no questionnaire respondent contributed data for more than one infant.

### 2.2. Outcome Measures

#### 2.2.1. Caregiver Outcomes

Parental psychological well-being was evaluated at enrollment and again at 6 and 12 months CA using three self-report measures.

Depressive symptoms were assessed using the official Korean version of the Beck Depression Inventory-II (K-BDI-II), a 21-item self-report measure with total scores ranging from 0 to 63, with higher scores indicating greater depressive symptom severity [6,7]. For descriptive screening analyses, a K-BDI-II score ≥ 17 was considered indicative of elevated depressive symptoms based on the optimal screening threshold for depressive disorders reported in a Korean adult validation study [7]. The Korean BDI-II demonstrated excellent internal consistency in that validation study (Cronbach’s α = 0.946) [7].

Parenting stress was assessed using the Korean Parenting Stress Index, Fourth Edition (K-PSI-4), full form [8,9]. The K-PSI-4 consists of 120 items, including the Child Domain (47 items), Parent Domain (54 items), and Life Stress items (19 items). The Child and Parent domains contribute to the Total Stress score. Questionnaires were scored using the standardized K-PSI-4 scoring system, and the resulting percentile scores for the Child Domain, Parent Domain, and Total Stress were used in the analyses, with higher percentile scores indicating greater parenting stress. The Korean standardization study reported high internal consistency for the Child Domain (α = 0.94), Parent Domain (α = 0.95), and Total Stress score (α = 0.97), with subscale coefficients ranging from 0.78 to 0.87 [9].

General self-efficacy was assessed using a 19-item Korean adaptation of the Self-Efficacy Scale originally developed by Sherer et al. [10,11]. The Korean adaptation comprises three subdomains: competency (8 items), confidence (6 items), and initiative (5 items). Items were rated on a 5-point Likert scale, with negatively worded items reverse-scored before calculation of the mean item score (range, 1–5); higher scores indicated greater general self-efficacy. Previous Korean use of this 19-item form reported good internal consistency (Cronbach’s α approximately 0.88) [11].

#### 2.2.2. Infant Outcomes

Infant temperament was assessed at enrollment, 6 months CA, and 12 months CA using the 191-item Korean translation of the Infant Behavior Questionnaire–Revised (IBQ-R) prepared by Keumjoo Kwak and Suchung Kim. Fourteen subscales were summarized into three higher-order dimensions—Surgency/Extraversion, Negative Affectivity, and Orienting/Regulation—according to the standard scoring procedure [12]. A separate Korean validation study of the 191-item IBQ-R supported its psychometric properties and reported Cronbach’s α values of approximately 0.67–0.92 across the temperament subscales [13].

Infant developmental outcomes were evaluated at 12 months CA using the Mental Development Index (MDI) and Psychomotor Development Index (PDI) of the Bayley Scales of Infant Development, Second Edition (BSID-II) [14].

Clinical variables, including gestational age, birth weight, sex, Apgar score, neonatal intensive care unit (NICU) length of stay, the interval between NICU discharge and enrollment, and neonatal brain injury, were obtained from the electronic medical records. Neonatal brain injury diagnoses and grades were extracted from clinically documented diagnoses; no central retrospective imaging re-review was performed for the present study. For exploratory analyses, infants were categorized as having no documented brain injury (n = 20), low-grade germinal matrix–intraventricular hemorrhage (GMH-IVH; grade 1–2; n = 7), or high-grade hemorrhagic injury (grade 3–4) and/or periventricular leukomalacia (PVL; n = 8). Because of the small number of infants in each diagnostic category, high-grade GMH-IVH and PVL were combined for exploratory analysis; this grouping was based on sample-size considerations rather than pathophysiological similarity. Family socioeconomic characteristics were collected using structured caregiver questionnaires.

### 2.3. Statistical Analysis

Continuous variables are presented as mean ± standard deviation (SD), and categorical variables as n (%). Two-sided *p* values < 0.05 and false discovery rate (FDR)-adjusted q values < 0.05 were considered statistically significant, as applicable. Analyses were performed using Python 3.13.5 (Python Software Foundation, Beaverton, OR, USA).

No formal a priori sample-size calculation or hierarchy of primary and secondary outcomes was prespecified. Given the exploratory design, the investigators pragmatically aimed to retain ≥30 dyads through 12 months. The eight domain-level questionnaire outcomes were treated as the main longitudinal analysis family; subscale, predictor–outcome, correlation, and brain-injury subgroup analyses were considered exploratory and hypothesis-generating.

The longitudinal analytic cohort comprised the 35 dyads retained through 12 months to characterize changes across the full follow-up period within a consistent cohort. Earlier observations from participants lost to contact were therefore excluded from the longitudinal analyses, and participants who withdrew consent were excluded from all analyses. Longitudinal changes in the eight domain-level outcomes (K-BDI-II; K-PSI-4 Total Stress, Parent Domain, Child Domain; general self-efficacy; and the three IBQ-R higher-order dimensions) were analyzed using linear mixed-effects models with subject-specific random intercepts and assessment wave as a categorical fixed effect. The eight overall time-effect tests were adjusted together using the Benjamini–Hochberg FDR procedure, and pairwise post hoc contrasts were Holm-adjusted. Because CA at enrollment varied among participants, sensitivity analyses for the three IBQ-R higher-order dimensions were additionally performed by including CA at enrollment as a covariate in the linear mixed-effects models. Exploratory subscale analyses were FDR-adjusted within each questionnaire instrument.

Changes in the proportion of parents with K-BDI-II scores ≥ 17 across the three assessments were evaluated using Cochran’s Q test among participants with data available at all three waves.

Associations between clinical, demographic, and socioeconomic characteristics and study outcomes were assessed using Pearson correlation coefficients or Cohen’s d, as appropriate, with separate FDR adjustment for baseline (104 tests) and 12-month (130 tests) analysis families. Concurrent caregiver-infant correlations at 12 months CA were assessed using Pearson or Spearman correlations, as appropriate based on normality assessed using the Shapiro–Wilk test, with FDR adjustment across 45 tests. Enrollment-to-12-month correlations constituted a separate exploratory family with FDR adjustment across 80 tests.

Neonatal brain-injury subgroup comparisons used Kruskal–Wallis tests, with FDR adjustment across the 10 overall outcome comparisons; pairwise Mann–Whitney U tests were Holm-adjusted.

Loss-to-follow-up-related selection bias was explored by comparing available baseline characteristics of participants retained through 12 months and those lost to contact using Welch’s *t* tests or Fisher’s exact tests, as appropriate.

Missing data were not imputed; linear mixed-effects models used all available repeated observations within this cohort, and other analyses used available cases.

## 3. Results

Within the 35-dyad analytic cohort, questionnaire completion varied across measures and study visits (Figure 1). Missing questionnaire data primarily resulted from missed visits or incomplete questionnaire responses.

To evaluate potential loss-to-follow-up bias, we compared available baseline characteristics between participants retained through 12 months (n = 35) and those lost to contact during follow-up (n = 18). No significant differences were identified in the available clinical, demographic, or psychological variables (Table S1, all *p* > 0.05). However, because participants who withdrew consent were not included in this comparison, the analysis does not assess potential selection related to consent withdrawal, and residual attrition-related selection bias cannot be excluded.

### 3.1. Baseline Characteristics of Preterm Infants and Caregivers

Within the 35-dyad analytic cohort, mean gestational age was 28.45 ± 2.93 weeks, and 16 infants (45.7%) were born before 28 weeks of gestation. Mean birth weight was 1114.8 ± 491.5 g. The baseline assessment was performed at a mean CA of 2.29 ± 1.31 months, ranging from 4 days before the estimated date of delivery to 5.0 months CA. Among infants with available NICU length-of-stay data (n = 32), the baseline assessment occurred 79.8 ± 42.5 days after the derived NICU discharge date (range, 28–180 days).

No documented neonatal brain injury was present in 20 infants (57.1%); 7 (20.0%) had grade 1–2 GMH-IVH, and 8 (22.9%) had grade 3–4 GMH/IVH and/or PVL.

Parents were identified as the primary day-to-day caregiver for 21 infants (60.0%), grandparents for 8 (22.9%), and babysitters for 4 (11.4%); this information was missing for 2 infants (5.7%). All questionnaire respondents were parents (33 mothers [94.3%] and 2 fathers [5.7%]), with the same parent remaining the respondent across all available assessment waves for each dyad. More than 80% of both mothers and fathers had completed at least a college education (Table 1).

### 3.2. Longitudinal Change in Caregiver Psychological Outcomes and Infant Temperament

Longitudinal changes in caregiver psychological measures and infant temperament from enrollment through 12 months CA are presented in Table 2. K-BDI-II scores decreased over time (overall *p* = 0.011, FDR q = 0.022), with significant model-estimated reductions from enrollment to 12 months and from 6 to 12 months after Holm adjustment. The proportion of parents with K-BDI-II scores ≥ 17 was 44.1% (15/34) at enrollment, 40.0% (12/30) at 6 months, and 30.3% (10/33) at 12 months CA. Among the 28 parents with K-BDI-II data at all three assessments, this change in proportion was not statistically significant (Cochran’s Q = 1.17, df = 2, *p* = 0.558).

K-PSI-4 Parent-domain stress also decreased over time (overall *p* = 0.004, FDR q = 0.011), with significant reductions from enrollment to 12 months and from 6 to 12 months. K-PSI-4 Total Stress, Child-domain stress, and general self-efficacy did not show significant longitudinal time effects after multiplicity adjustment.

Infant Surgency/Extraversion increased over time (overall *p* < 0.001, FDR q < 0.008), with significant pairwise increases across all three assessment intervals. Negative Affectivity also increased over time (overall *p* = 0.002, FDR q = 0.008), with a significant increase from enrollment to 12 months CA. Orienting/Regulation did not show a significant overall longitudinal change (*p* = 0.098, FDR q = 0.157). Subscale findings are presented in Supplementary Table S2.

Adjustment for CA at enrollment did not materially alter the longitudinal findings for the three IBQ-R higher-order dimensions. Overall time effects remained significant for Surgency/Extraversion (*p* < 0.001) and Negative Affectivity (*p* = 0.003), whereas Orienting/Regulation remained nonsignificant (*p* = 0.097). CA at enrollment itself was not significantly associated with any of the three higher-order dimensions in these sensitivity models (all *p* > 0.20; Supplementary Table S3).

### 3.3. Exploratory Associations Between Clinical or Socioeconomic Characteristics and Study Outcomes

Exploratory associations between clinical, demographic, and socioeconomic characteristics and study outcomes are reported in Supplementary Tables S4 and S5. At enrollment, 4 of 104 comparisons reached nominal significance, and 13 of 130 comparisons reached nominal significance at 12 months CA. None remained statistically significant after FDR correction within the corresponding analysis families. Given the limited sample size and extensive multiplicity, these findings were considered inconclusive and hypothesis-generating.

Exploratory comparisons according to neonatal brain injury category are presented in Supplementary Table S6. A nominal between-group difference was observed for IBQ-R Orienting/Regulation (unadjusted *p* = 0.025); however, this finding did not remain statistically significant after FDR correction across the 10 subgroup outcome comparisons (q = 0.250). No other outcome differed significantly after multiplicity adjustment.

### 3.4. Correlations Among Caregiver and Infant Outcomes

Correlations among caregiver psychological measures, infant temperament, and developmental outcomes at 12 months CA are reported in Supplementary Table S7 and visualized in Supplementary Figure S1. Several within-domain correlations remained statistically significant after FDR correction, including correlations among caregiver psychological measures and between selected infant developmental or temperament measures.

Among the domain-level cross-domain analyses, K-PSI-4 Total Stress was positively correlated with parent-reported IBQ-R Negative Affectivity (r = 0.47, FDR q = 0.039). At the subscale level, several exploratory correlations between K-PSI-4 and IBQ-R subscales also remained statistically significant after FDR correction (Supplementary Table S8). These analyses were considered exploratory and hypothesis-generating.

### 3.5. Exploratory Longitudinal Associations Between Enrollment and 12-Month Outcomes

Enrollment-to-12-month correlations are presented in Supplementary Table S9. Several correlations reached nominal statistical significance, but none remained statistically significant after FDR correction across the 80 longitudinal correlation tests. These findings were therefore considered inconclusive rather than evidence for weak or absent longitudinal relationships.

## 4. Discussion

### 4.1. Main Findings

The present prospective longitudinal study characterized changes in caregiver psychological measures and infant temperament from enrollment through 12 months CA following preterm birth while exploring their clinical correlates and interrelationships. Three main longitudinal findings were observed. First, caregiver depressive symptom scores and Parent-domain parenting stress decreased over time, whereas general self-efficacy did not show a significant longitudinal change. Second, infant Surgency/Extraversion and Negative Affectivity increased over time, whereas Orienting/Regulation did not show a significant longitudinal change. These temperament findings were materially unchanged after adjustment for CA at enrollment. Third, most exploratory domain-level cross-domain correlations did not remain statistically significant after FDR correction; with the exception of a positive correlation between K-PSI-4 Total Stress and parent-reported infant Negative Affectivity.

### 4.2. Longitudinal Changes in Caregiver Psychological Well-Being

Caregiver depressive symptom scores decreased over time, particularly between 6 and 12 months CA. This pattern is consistent with previous studies reporting decreases in psychological distress among parents of preterm infants over time [15,16]. Parent-domain parenting stress also decreased [17], whereas Child-domain and Total Stress scores did not show significant longitudinal changes. Although these findings may reflect changes in caregiver psychological well-being over time, the underlying mechanisms, including possible adaptation to the parenting role, cannot be determined from the present study.

Exploratory subscale analyses showed decreases in Attachment and Reinforces Parent scores, along with changes in several other K-PSI-4 subscales. Although these score changes may reflect shifts in specific dimensions captured by the instrument, they should not be interpreted as direct evidence of stronger emotional bonding or increasingly rewarding parent–infant interactions. Distractibility/Hyperactivity increased over time and may be consistent with age-related changes in infant behavior as children became older and increasingly mobile and exploratory; however, the present study cannot distinguish developmental maturation from other potential influences on these changes.

General self-efficacy did not show a significant longitudinal change. Because the instrument used in this study assessed general rather than parenting-specific self-efficacy, this finding should not be interpreted as evidence regarding parenting competence or parenting-specific efficacy. Taken together, the differing longitudinal patterns across depressive symptoms, parenting-stress domains, and general self-efficacy highlight that these questionnaires assess distinct aspects of caregiver psychological experience.

### 4.3. Longitudinal Changes in Infant Temperament

Infant temperament showed longitudinal changes from enrollment through 12 months CA. Surgency/Extraversion increased steadily over time, with corresponding increases in several exploratory subscales, including Approach, High-Intensity Pleasure, Perceptual Sensitivity, Smiling and Laughter, and Vocal Reactivity. Negative Affectivity also increased, with corresponding increases in Distress to Limitation and Fear. These patterns may be consistent with age-related behavioral and emotional changes during infancy; however, their developmental origin cannot be established from the present within-cohort design.

Orienting/Regulation did not show a significant overall longitudinal change after multiplicity adjustment. Importantly, adjustment for CA at enrollment did not materially alter the longitudinal results for any of the three IBQ-R higher-order dimensions. This suggests that variation in CA at enrollment did not account for the observed time-effect pattern within this cohort. Nevertheless, because the study did not include a term-born comparison group or apply normative longitudinal trajectories, the observed changes cannot be interpreted as direct evidence of typical developmental maturation.

Thus, the present findings demonstrate within-cohort longitudinal changes in selected dimensions of parent-reported infant temperament but do not establish the developmental mechanisms underlying those changes.

### 4.4. Exploratory Associations of Baseline Characteristics with Study Outcomes

Several nominal associations were observed between neonatal, demographic, and socioeconomic characteristics and study outcomes, but none remained statistically significant after FDR correction within the corresponding analysis families. Given the limited sample size, extensive multiplicity, and resulting uncertainty in the effect estimates, these findings should be considered inconclusive and hypothesis-generating rather than evidence that the underlying associations are weak or absent. Caregiver psychological well-being and infant development may be influenced by multiple biological, environmental, and family-level factors; however, the present exploratory analyses were not sufficiently powered to determine the relative contributions of individual characteristics. Larger multicenter cohorts will be required to estimate these associations with greater precision.

In exploratory analyses according to neonatal brain injury category, Orienting/Regulation showed a nominal between-group difference (unadjusted *p* = 0.025), but this finding did not remain significant after FDR correction across the 10 outcomes examined (q = 0.250). MDI and PDI scores were numerically lower in the group containing higher-grade hemorrhagic injury and/or PVL, but these comparisons likewise did not remain statistically significant after multiplicity adjustment. Given the small subgroup sizes and the clinical and pathophysiological heterogeneity of the grouped brain-injury diagnoses, no firm conclusion regarding associations between neonatal brain injury category and the measured behavioral or developmental outcomes can be drawn from the present data [18,19].

### 4.5. Caregiver-Infant Associations and Longitudinal Patterns of Caregiver Psychological Measures

Several within-domain correlations remained statistically significant after FDR correction, whereas most domain-level cross-domain correlations did not. Given the limited sample size and extensive multiplicity, the latter findings should be regarded as inconclusive rather than as evidence that caregiver psychological well-being and infant characteristics are weakly related or uncoupled. More complex reciprocal processes remain possible within the transactional model of development, in which caregiver and child characteristics may influence one another over time [20,21].

Among the domain-level cross-domain analyses, K-PSI-4 Total Stress was positively correlated with parent-reported IBQ-R Negative Affectivity after FDR correction. However, because both instruments were completed by the same parent, this association may partly reflect shared-reporter or common-method variance, as parental psychological state or perceived caregiving difficulty may influence the reporting of both parenting stress and infant behavior. Therefore, this cross-sectional association should not be interpreted as evidence of a directional or causal relationship between parenting stress and infant temperament. Previous studies have similarly suggested associations between parental psychological functioning and aspects of infant temperament or parent–child interaction [22,23], but multimethod assessments using independent reporters are needed to clarify these relationships.

Several exploratory K-PSI-4–IBQ-R subscale correlations also remained statistically significant after FDR correction. These findings identify specific questionnaire dimensions for further investigation but should be considered hypothesis-generating rather than evidence of established caregiver–infant interaction mechanisms, particularly because both instruments were completed by the same parent.

General self-efficacy showed a different pattern of correlations from depressive symptoms and parenting stress, consistent with its assessment of a broader self-efficacy construct rather than parenting-specific competence.

In addition to these cross-sectional caregiver–infant associations, several moderate enrollment-to-12-month correlations were observed for caregiver psychological measures, including K-BDI-II scores (r = 0.55) and K-PSI-4 Parent-domain scores (r = 0.50). However, none remained significant after FDR correction across 80 longitudinal correlation tests. Given the limited sample size and multiple testing, these findings are insufficient to establish the longitudinal stability of caregiver psychological measures.

### 4.6. Strengths, Limitations, and Clinical Implications

This study has several strengths. It prospectively followed preterm infant–parent dyads with repeated assessments of caregiver psychological well-being and infant temperament through 12 months CA. The same parent served as the questionnaire respondent across all available assessment waves for each dyad, supporting within-respondent comparability over time. Concurrent assessment of depressive symptoms, parenting stress, general self-efficacy, temperament, and neurodevelopment allowed multiple dimensions of caregiver and infant functioning to be examined within the same longitudinal cohort. In addition, sensitivity analyses accounting for CA at enrollment yielded longitudinal temperament findings consistent with the main analyses.

From a family-level perspective, the Family Stress Model provides a useful framework for understanding caregiver psychological well-being after preterm birth, emphasizing how contextual stressors and caregiver psychological distress may influence broader family processes relevant to child adjustment [5]. Repeated assessment may therefore help identify parents experiencing persistent or emerging psychological distress during follow-up. Importantly, screening alone does not constitute an intervention. When clinically significant distress or caregiving difficulties are identified, follow-up programs may consider referral for appropriate psychological support, parent education, or family-centered early intervention. Recent systematic reviews and meta-analyses suggest that parenting, educational, and psychological interventions for parents of preterm or NICU-admitted infants may improve selected parental outcomes, including self-efficacy, stress, anxiety, and depressive symptoms [24,25,26]. However, because the present study was observational and did not evaluate an intervention, the effectiveness of such approaches cannot be inferred from our findings.

Several limitations should be acknowledged. First, the relatively small longitudinal analytic cohort and single-center design limited statistical power and the precision of effect estimates. Although FDR correction was used to reduce the risk of false-positive findings arising from multiple comparisons, multiplicity adjustment does not compensate for limited power or imprecise estimates in a small sample. Accordingly, the association, correlation, subscale, and subgroup analyses should be considered exploratory and hypothesis-generating and require confirmation in larger independent cohorts. In addition, recruitment from a pediatric rehabilitation outpatient clinic may have overrepresented preterm infants with greater medical or developmental concerns, while the relatively high educational attainment of participating parents may limit generalizability to families with different socioeconomic and educational backgrounds.

Second, the longitudinal analysis was restricted to dyads retained through 12 months. This cohort-based restriction meant that earlier available observations from participants subsequently lost to contact were not included in the longitudinal models, reducing the amount of available data and raising the possibility of attrition-related selection bias. Available baseline characteristics did not differ significantly between participants retained through 12 months and those lost to contact; however, the small number of participants lost to contact and incomplete data for some baseline variables limit this comparison, and residual selection bias cannot be excluded. Participants who withdrew consent were not included in this comparison.

Third, several measurement-related limitations should be considered. Parenting stress and infant temperament were reported by the same parent, and associations between K-PSI-4 and IBQ-R scores may therefore be influenced by shared-reporter or common-method variance. The absence of a term-born comparison group or normative longitudinal reference data limits our ability to distinguish observed within-cohort changes in infant temperament from typical age-related developmental changes. Some IBQ-R assessments at enrollment were performed before 3 months CA, younger than the age range represented in the Korean validation study, and should therefore be interpreted cautiously. The self-efficacy measure assessed general rather than parenting-specific self-efficacy. In addition, item-level questionnaire responses were not available in the analytic dataset, precluding estimation of sample-specific internal consistency. Reliability estimates from previous validation studies therefore provide relevant psychometric context but cannot substitute for sample-specific reliability estimates in the present cohort.

Finally, BSID-II developmental outcomes were available for only 26 infants, limiting the precision and generalizability of analyses involving developmental outcomes. Follow-up through 12 months CA also precluded evaluation of longer-term caregiver and infant outcomes.

## 5. Conclusions

In conclusion, caregiver psychological measures and infant temperament showed longitudinal changes from enrollment to 12 months CA following preterm birth. Depressive symptoms and Parent-domain parenting stress decreased over time, whereas infant Surgency/Extraversion and Negative Affectivity increased. Most exploratory cross-domain associations did not remain significant after multiplicity adjustment. These within-cohort findings require confirmation in larger, more diverse longitudinal cohorts, and repeated assessment of caregiver psychological well-being may complement developmental follow-up of preterm infants.

## Figures and Tables

**Figure 1:**
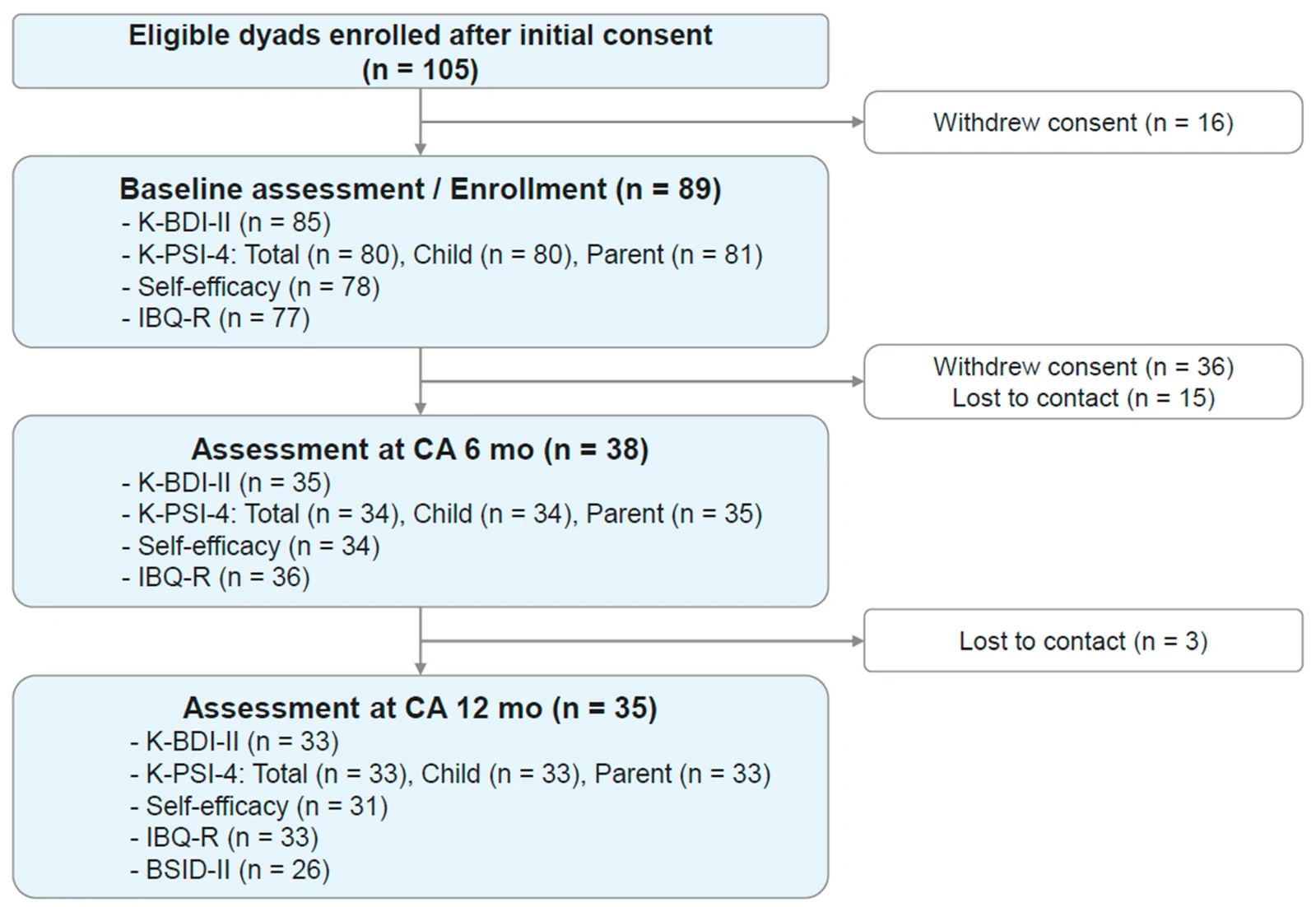
Study flow, assessment schedule, and availability of study measures. CA, corrected age; K-BDI-II, Korean Beck Depression Inventory-II; K-PSI-4, Korean Parenting Stress Index, Fourth Edition; IBQ-R, Infant Behavior Questionnaire-Revised; BSID-II, Bayley Scales of Infant Development, Second Edition.

**Table 1:** Baseline demographic and clinical characteristics of preterm infants and their primary caregivers.

Characteristic	Value
**Infant factors**	
Gestational age, weeks	28.45 ± 2.93
<28 weeks:28 to <32 weeks:≥32 weeks	16 (45.7%):13 (37.1%):6 (17.1%)
Birth weight, g	1114.80 ± 491.49
<1000 g:1000 to <1500 g:≥1500 g	18 (51.4%):8 (22.9%):9 (25.7%)
Female sex	15 (42.9%)
Apgar score at 5 min	6.55 ± 1.62 (n = 29)
Neonatal brain injury category (None:grade 1–2 GMH-IVH:grade 3–4 GMH/IVH and/or PVL)	20 (57.1%):7 (20.0%):8 (22.9%)
NICU stay, days	75.94 ± 36.68 (n = 32)
Interval from NICU discharge to baseline assessment (days)	79.8 ± 42.5 (n = 32; range, 28–180)
Corrected age at baseline assessment (months)	2.29 ± 1.31 (range, −0.13 to 4.93)
MDI of BSID-II at CA 12 months	88.92 ± 12.73 (n = 26)
PDI of BSID-II at CA 12 months	82.77 ± 14.11 (n = 26)
**Caregiver/family factors**	
Questionnaire respondent (Mother:Father)	33 (94.3%):2 (5.7%)
Maternal age (years)	35.73 ± 3.31 (n = 33)
Maternal education (≤High school:College:Graduate school:Missing)	4 (11.4%):23 (65.7%):6 (17.1%):2 (5.7%)
Paternal education (≤High school:College:Graduate school:Missing)	3 (8.6%):29 (82.9%):1 (2.9%):2 (5.7%)
Household income (<$37.5k:$37.5k–75k:>$75k:Missing)	6 (17.1%):15 (42.9%):12 (34.3%):2 (5.7%)
Dual-income family	17 (48.6%)
Siblings present	17 (48.6%)
Maternal medical conditions (Yes:No:Missing)	7 (20.0%):26 (74.3%):2 (5.7%)
Primary day-to-day caregiver (Parent:Grandparent:Babysitter:Missing)	21 (60.0%):8 (22.9%):4 (11.4%):2 (5.7%)

Continuous variables are mean ± SD (n shown when <35); categorical variables are n (%) of the full cohort. CA: Corrected age; GMH-IVH: Germinal matrix hemorrhage-intraventricular hemorrhage; PVL: Periventricular leukomalacia; NICU: Neonatal intensive care unit; MDI: Mental Developmental Index; PDI: Psychomotor Developmental Index; BSID-II: Bayley Scales of Infant Development, Second Edition.

**Table 2:** Longitudinal changes in caregiver psychological well-being and infant temperament from enrollment through 12 months corrected age.

Outcome	Observed Mean ± SD (n)			Overall *p*	FDR q	Model-Estimated Changes			
	Enrollment	6 Months CA	12 Months CA			Comparison	Change	95% CI	Holm *p*
K-BDI-II Total	16.76 ± 8.51 (n = 34)	16.80 ± 8.38 (n = 30)	13.58 ± 7.36 (n = 33)	0.011 *	**0.022 †**	6 mo − enroll.	−0.49	−2.80 to 1.82	0.679
						12 mo − enroll.	−3.23	−5.48 to −0.98	**0.015 ‡**
						12 mo − 6 mo	−2.74	−5.10 to −0.39	**0.045 ‡**
K-PSI-4 Total	51.02 ± 27.47 (n = 32)	48.78 ± 25.09 (n = 28)	41.86 ± 22.47 (n = 33)	0.156	0.208	6 mo − enroll.	−2.51	−10.86 to 5.84	0.556
						12 mo − enroll.	−7.81	−15.90 to 0.27	0.174
						12 mo − 6 mo	−5.30	−13.78 to 3.18	0.440
K-PSI-4 Child	39.60 ± 27.04 (n = 32)	37.02 ± 25.55 (n = 28)	38.63 ± 20.53 (n = 33)	0.710	0.710	6 mo − enroll.	−2.98	−11.47 to 5.51	1.000
						12 mo − enroll.	+0.33	−7.87 to 8.54	1.000
						12 mo − 6 mo	+3.32	−5.29 to 11.92	1.000
K-PSI-4 Parent	58.11 ± 29.12 (n = 33)	59.24 ± 28.00 (n = 29)	45.61 ± 25.68 (n = 33)	0.004 *	**0.011 †**	6 mo − enroll.	−0.76	−8.70 to 7.17	0.850
						12 mo − enroll.	−11.86	−19.57 to −4.16	**0.008 ‡**
						12 mo − 6 mo	−11.10	−19.18 to −3.01	**0.014 ‡**
Self-Efficacy	3.05 ± 0.17 (n = 32)	2.99 ± 0.14 (n = 26)	3.04 ± 0.18 (n = 31)	0.369	0.422	6 mo − enroll.	−0.05	−0.14 to 0.03	0.574
						12 mo − enroll.	−0.01	−0.08 to 0.07	0.872
						12 mo − 6 mo	+0.05	−0.03 to 0.13	0.574
IBQ-R Surgency/Extraversion	3.64 ± 0.72 (n = 35)	4.26 ± 0.67 (n = 30)	4.65 ± 0.68 (n = 33)	<0.001 *	**<0.008 †**	6 mo − enroll.	+0.62	0.37 to 0.88	**<0.001 ‡**
						12 mo − enroll.	+0.98	0.73 to 1.23	**<0.001 ‡**
						12 mo − 6 mo	+0.36	0.09 to 0.62	**0.008 ‡**
IBQ-R Negative Affectivity	3.21 ± 0.70 (n = 35)	3.45 ± 0.68 (n = 30)	3.60 ± 0.61 (n = 33)	0.002 *	**0.008 †**	6 mo − enroll.	+0.21	−0.01 to 0.44	0.128
						12 mo − enroll.	+0.38	0.16 to 0.60	**0.002 ‡**
						12 mo − 6 mo	+0.17	−0.06 to 0.40	0.155
IBQ-R Orienting/Regulation	4.77 ± 0.78 (n = 35)	5.01 ± 0.64 (n = 30)	5.07 ± 0.51 (n = 33)	0.098	0.157	6 mo − enroll.	+0.24	−0.05 to 0.52	0.201
						12 mo − enroll.	+0.28	0.01 to 0.55	0.136
						12 mo − 6 mo	+0.04	−0.24 to 0.33	0.766

Values are observed mean ± SD (n). Estimated changes and 95% confidence intervals (CIs) are fixed-effect contrasts from random-intercept linear mixed-effects models using all available observations within the 35-dyad longitudinal analytic cohort. Overall *p*-values are 2-df Wald tests of the time effect. FDR q-values are adjusted across the eight overall time-effect tests comprising the main longitudinal analysis family using the Benjamini–Hochberg procedure. Pairwise contrast *p*-values (Holm *p*) are adjusted using the Holm method. Changes represent the later minus the earlier assessment. Bold *p*- and q-values indicate statistical significance (<0.05). * Overall *p* < 0.05; † FDR q < 0.05; ‡ Holm-adjusted pairwise *p* < 0.05. CA, corrected age; SD, standard deviation; FDR, false discovery rate; enroll., enrollment; K-BDI-II, Korean Beck Depression Inventory-II; K-PSI-4, Korean Parenting Stress Index, Fourth Edition; IBQ-R, Infant Behavior Questionnaire–Revised. Self-efficacy refers to general self-efficacy.

## Data Availability

The data supporting the findings of this study are not publicly available due to privacy and ethical restrictions related to participant confidentiality but are available from the corresponding author upon reasonable request.

## Supplementary Materials

The following supporting information can be downloaded at: https://www.mdpi.com/article/10.3390/children13101295/s1, Figure S1: Correlations among caregiver psychological well-being, infant temperament, and developmental outcomes at 12 months corrected age. Pearson’s correlation coefficient (r) or Spearman’s rank correlation coefficient (ρ) was used, as appropriate. Correlation coefficients marked with † and outlined in thick black remained significant after Benjamini–Hochberg false discovery rate (FDR) correction across the 45 predefined pairwise comparisons. The selected correlation coefficient and pairwise sample size are reported in Supplementary Table S7. K-BDI-II: Korean Beck Depression Inventory-II; IBQ-R: Infant Behavior Questionnaire–Revised; K-PSI-4: Korean Parenting Stress Index, Fourth Edition; MDI: Mental Developmental Index; PDI: Psychomotor Developmental Index; Table S1: Comparison of available baseline characteristics between participants retained through 12-month follow-up and those lost to contact; Table S2: Longitudinal changes in K-PSI-4, self-efficacy, and IBQ-R subscales; Table S3: Sensitivity analyses of longitudinal infant temperament outcomes adjusted for corrected age at enrollment; Table S4: Associations between baseline clinical characteristics and study outcomes at enrollment; Table S5: Associations between clinical characteristics and study outcomes at 12 months corrected age; Table S6: Caregiver psychological, infant developmental and behavioral outcomes at 12 months according to neonatal brain injury category; Table S7: Correlations among caregiver psychological well-being, infant temperament, and developmental outcomes at 12 months corrected age; Table S8: Significant cross-domain correlations among questionnaire subscales at 12 months; Table S9: Longitudinal correlations between enrollment and 12-month outcomes.

## References

[1] Behrman R.E., Butler A.S., Institute of Medicine (US) Committee on Understanding Premature Birth and Assuring Healthy Outcomes (2007). Preterm Birth: Causes, Consequences, and Prevention.

[2] Johnson S., Marlow N. (2011). Preterm birth and childhood psychiatric disorders. Pediatr. Res..

[3] Singh G.K., Kenney M.K., Ghandour R.M., Kogan M.D., Lu M.C. (2013). Mental health outcomes in US children and adolescents born prematurely or with low birthweight. Depress. Res. Treat..

[4] Pace C.C., Spittle A.J., Molesworth C.M.-L., Lee K.J., Northam E.A., Cheong J.L.Y., Davis P.G., Doyle L.W., Treyvaud K., Anderson P.J. (2016). Evolution of depression and anxiety symptoms in parents of very preterm infants during the newborn period. JAMA Pediatr..

[5] Masarik A.S., Conger R.D. (2017). Stress and child development: A review of the Family Stress Model. Curr. Opin. Psychol..

[6] Beck A.T., Steer R.A., Brown G.K. (1996). Manual for the Beck Depression Inventory-II.

[7] Park K., Jaekal E., Yoon S., Lee S.-H., Choi K.-H. (2020). Diagnostic utility and psychometric properties of the Beck Depression Inventory-II among Korean adults. Front. Psychol..

[8] Abidin R.R. (2012). PSI-4 Professional Manual.

[9] Chung K., Lee S.I., Lee C. (2019). Standardization study for the Korean version of Parenting Stress Index Fourth Edition (K-PSI-4). Korean J. Psychol. Gen..

[10] Sherer M., Maddux J.E., Mercandante B., Prentice-Dunn S., Jacobs B., Rogers R.W. (1982). The Self-Efficacy Scale: Construction and validation. Psychol. Rep..

[11] Park J.I. (2008). The Moderating Effects of Spiritual Well-Being on Self-Efficacy and Job Satisfaction of the Social Workers. Doctoral Dissertation.

[12] Gartstein M.A., Rothbart M.K. (2003). Studying infant temperament via the Revised Infant Behavior Questionnaire. Infant Behav. Dev..

[13] Lim J., Bae Y.J. (2021). Validation study of the Korean version of Rothbart’s Infant Behavior Questionnaire-Revised. Fam. Environ. Res..

[14] Bayley N. (1993). Bayley Scales of Infant Development.

[15] Sandnes R., Le Floch M., Riquin E., Nocus I., Müller J.B., Bacro F. (2024). Parental stress and mental health outcomes following very preterm birth: A systematic review of recent findings. J. Affect. Disord..

[16] Nguyen C.T.T., Sandhi A., Lee G.T., Nguyen L.T.K., Kuo S.Y. (2023). Prevalence of and factors associated with postnatal depression and anxiety among parents of preterm infants: A systematic review and meta-analysis. J. Affect. Disord..

[17] Gray P.H., Edwards D.M., O’Callaghan M.J., Cuskelly M., Gibbons K. (2013). Parenting stress in mothers of very preterm infants—Influence of development, temperament and maternal depression. Early Hum. Dev..

[18] Cha J.H., Choi N., Kim J., Lee H.J., Na J.Y., Park H.K. (2022). Cystic periventricular leukomalacia worsens developmental outcomes of very-low-birth-weight infants with intraventricular hemorrhage: A nationwide cohort study. J. Clin. Med..

[19] Woodward L.J., Clark C.A.C., Bora S., Inder T.E. (2012). Neonatal white matter abnormalities an important predictor of neurocognitive outcome for very preterm children. PLoS ONE.

[20] Sameroff A.J., MacKenzie M.J. (2003). Research strategies for capturing transactional models of development: The limits of the possible. Dev. Psychopathol..

[21] Sameroff A. (2010). A unified theory of development: A dialectic integration of nature and nurture. Child Dev..

[22] Mattera J.A., Campagna A.X., Goodman S.H., Gartstein M.A., Hancock G.R., Stowe Z.N., Newport D.J., Knight B.T. (2023). Associations between mothers’ and fathers’ depression and anxiety prior to birth and infant temperament trajectories over the first year of life: Evidence from diagnoses and symptom severity. J. Affect. Disord..

[23] Gerstein E.D., Poehlmann-Tynan J. (2015). Transactional processes in children born preterm: Influences of mother–child interactions and parenting stress. J. Fam. Psychol..

[24] Dewi D.T.K., Nguyen C.T.T., Chen S.-R., Lee G.T., Tsai S.-Y., Huda M.H., Kuo S.-Y. (2025). Effectiveness of parenting interventions on self-efficacy, anxiety, stress, and depression among parents of preterm infants: A systematic review and meta-analysis of randomized control trials. Int. J. Nurs. Stud..

[25] Padbidri P., Joshi V., Hix-Small H., Dekhane P.B. (2026). Impact of parent education programmes on confidence and self-efficacy in parents of preterm infants: A systematic review. BMJ Paediatr. Open.

[26] Niehaus Milligan C.E., Hayes Austin L.R., Auckland P., Hatfield O., Bills S.E., Daffin M., Keith J., Genova G., Black A., Tsujimoto K. (2026). Parent Mental Health Interventions in the NICU: A systematic review and meta-analysis. JAMA Pediatr..

