# Supplementary material for: Longitudinal Changes in Caregiver Psychological Well-Being and Infant Temperament Following Preterm Birth: From Enrollment to 12 Months Corrected Age
Source: Children (Basel). 2026 Sep 24;13(10):1295. doi: 10.3390/children13101295 (PMC13703861; doi:10.3390/children13101295)
Supplement: Supplementary file 1 [file children-13-01295-s001.zip › children-4526131-supplementary.pdf]

Supplementary Material

Figure S1. Correlations among caregiver psychological well-being, infant temperament, and developmental outcomes at 12 months corrected age. Pearson's correlation coefficient (r) or Spearman's rank correlation coefficient (ρ) was used, as appropriate. Correlation coefficients marked with † and outlined in thick black remained significant after Benjamini–Hochberg false discovery rate (FDR) correction across the 45 predefined pairwise comparisons. The selected correlation coefficient and pairwise sample size are reported in Supplementary Table S7. K-BDI-II: Korean Beck Depression Inventory-II; IBQ-R: Infant Behavior Questionnaire–Revised; K-PSI-4: Korean Parenting Stress Index, Fourth Edition; MDI: Mental Developmental Index; PDI: Psychomotor Developmental Index.

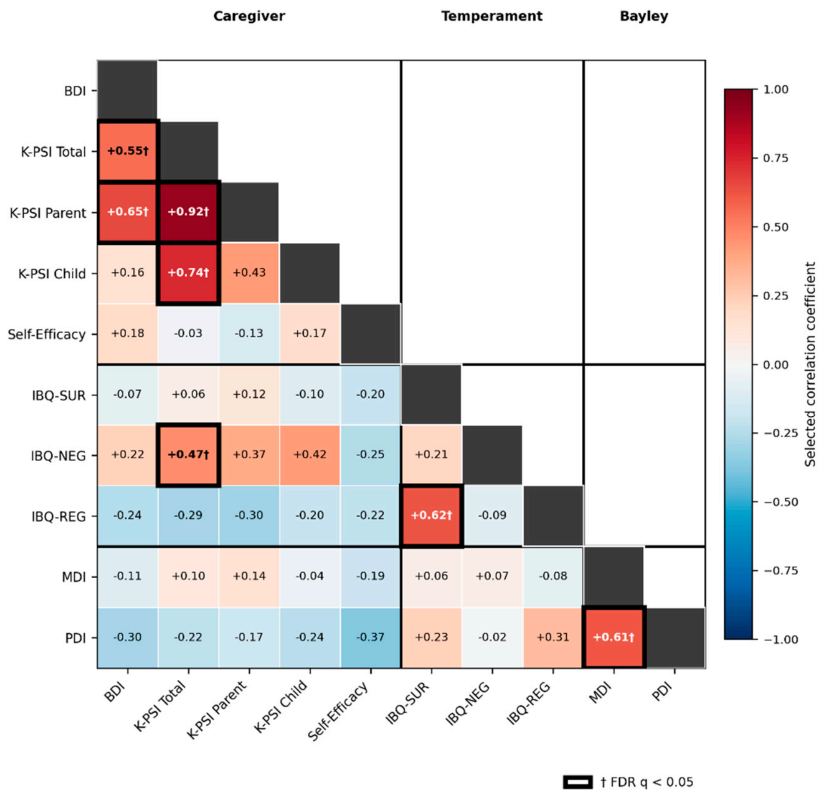

Caregiver
Temperament
Bayley
BDI
K-PSI Total
K-PSI Parent
K-PSI Child
Self-Efficacy
IBQ-SUR
IBQ-NEG
IBQ-REG
MDI
PDI
Selected correlation coefficient
† FDR q < 0.05

Table S1. Comparison of available baseline characteristics between participants retained through 12-month follow-up and those lost to contact.

Baseline variable	Retained through 12 months	Lost to contact	p value
**Gestational age, weeks**	28.45 ± 2.93 (n = 35)	30.18 ± 3.36 (n = 18)	0.074
**Birth weight, g**	1115 ± 491 (n = 35)	1131 ± 415 (n = 18)	0.900
**Female sex, n/N (%)**	15/35 (42.9%)	8/18 (44.4%)	1.000
**Apgar score at 5 min**	6.55 ± 1.62 (n = 29)	7.47 ± 2.00 (n = 17)	0.119
**Maternal age, years**	35.73 ± 3.31 (n = 33)	37.06 ± 3.78 (n = 18)	0.220
**Baseline K-BDI-II**	16.76 ± 8.51 (n = 34)	16.33 ± 8.57 (n = 18)	0.864
**Baseline K-PSI-4 Total**	51.02 ± 27.47 (n = 32)	58.17 ± 26.82 (n = 17)	0.384
**Baseline Self-efficacy**	3.05 ± 0.17 (n = 32)	3.00 ± 0.23 (n = 15)	0.487

Values are mean ± SD (number of participants with available data). Participants who withdrew consent (n = 36) were excluded from this loss-to-follow-up analysis. Comparisons were based on participants with available baseline data for each variable; therefore, sample sizes may vary across comparisons.

Table S2. Longitudinal changes in K-PSI-4, Self-efficacy, and IBQ-R subscales.

Instrument	Subscale		Enrollment	6 months	12 months	Overall p	FDR q
**K-PSI-4**	**Child Domain**	Distractibility/Hyperactivity (DI)	39.13±26.05	48.32±33.46	58.92±25.02	<0.001*	0.0023†
		Adaptability (AD)	49.02±29.57	46.39±28.19	47.82±26.34	0.7833	0.8486
		Reinforces Parent (RE)	35.98±29.10	24.59±19.66	17.41±15.83	<0.001*	0.0023†
		Demandingness (DE)	50.12±32.07	39.01±24.82	40.05±23.80	0.0537	0.1089
		Mood (MO)	33.47±29.00	27.44±22.64	29.12±24.30	0.3391	0.4409
		Acceptability (AC)	36.28±24.53	41.41±28.29	40.55±23.75	0.6167	0.7288
	**Parent Domain**	Competence (CO)	58.75±30.37	56.11±29.98	47.64±27.57	0.0428*	0.1089
		Isolation (IS)	53.16±28.06	59.40±29.11	53.36±26.46	0.2438	0.3522
		Attachment (AT)	39.75±30.74	37.63±29.41	25.85±19.87	0.0011*	0.0049†
		Health (HE)	62.30±31.39	61.85±29.76	61.02±32.00	0.8969	0.8969
		Role Restriction (RO)	77.78±22.64	78.13±24.26	64.80±27.06	0.0026*	0.0086†
		Depression (DP)	51.06±30.68	53.77±32.69	43.03±29.29	0.0586	0.1089
		Spouse/Parenting Partner Relationship (SP)	37.66±26.99	41.81±29.06	31.43±23.79	0.1080	0.1756
**Self-efficacy**	**Competency**		2.22±0.51	2.26±0.46	2.24±0.51	0.7558	0.7863
	**Confidence**		3.85±0.56	3.60±0.67	3.79±0.61	0.2999	0.7863
	**Initiative**		3.34±0.48	3.28±0.27	3.34±0.45	0.7863	0.7863
**IBQ-R**	**Surgency/Extraversion**	Activity Level (ACT)	3.43±1.08	3.78±0.93	3.63±0.72	0.2189	0.3405
		Approach (APP)	3.57±1.56	4.39±1.19	4.99±1.02	<0.001*	<0.001†
		High-Intensity Pleasure (HIP)	4.56±1.32	5.47±1.08	6.12±0.72	<0.001*	<0.001†
		Perceptual Sensitivity (PER)	3.23±0.99	3.88±1.03	4.57±0.97	<0.001*	<0.001†
		Smiling and Laughter (SMI)	3.79±1.17	4.40±0.92	4.57±1.03	<0.001*	0.0019†
		Vocal Reactivity (VOC)	3.27±0.96	3.63±0.90	4.03±1.01	<0.001*	0.0019†
	**Negative Affectivity**	Distress to Limitations (DTL)	3.63±0.98	3.60±0.73	3.99±0.72	0.0162*	0.0284†
		Fear (FEA)	2.96±1.37	3.57±1.16	4.01±0.91	<0.001*	<0.001†
		Falling Reactivity (FAL, reverse-scored)	4.95±0.86	4.79±1.13	5.03±0.83	0.4853	0.5227
		Sadness (SAD)	3.16±0.91	3.35±1.03	3.44±0.90	0.4044	0.4718

**Orienting/Regulation**	Cuddliness (CUD)	5.64±0.59	5.63±0.51	5.60±0.58	0.8664	0.8664
	Duration of Orienting (DUR)	4.05±1.54	4.38±1.29	4.37±1.09	0.3565	0.4537
	Low-Intensity Pleasure (LIP)	4.59±1.43	5.13±0.89	5.23±0.82	0.0130*	0.0259†
	Soothability (SOO)	4.81±0.81	4.90±0.89	5.08±0.60	0.3046	0.4265

Values are mean ± SD. * Unadjusted/overall $p < 0.05$ by linear mixed-effects models; † FDR-adjusted $q < 0.05$ for K-PSI-4, self-efficacy, and IBQ-R subscales using the Benjamini-Hochberg procedure. IBQ-R: Infant Behavior Questionnaire-Revised; K-PSI-4: Korean Parenting Stress Index, Fourth Edition.

Subscale analyses were exploratory.

Table S3. Sensitivity analyses of longitudinal infant temperament outcomes adjusted for corrected age at enrollment.

Outcome	Main model overall time p	CA-adjusted overall time p	Baseline corrected age covariate p
**IBQ-R (Surgency/Extraversion)**	<0.001*	<0.001*	0.221
**IBQ-R (Negative Affectivity)**	0.002*	0.003*	0.335
**IBQ-R (Orienting/Regulation)**	0.098	0.097	0.801

Sensitivity models included assessment wave and corrected age at enrollment as fixed effects with a subject-specific random intercept. CA: Corrected Age; IBQ-R: Infant Behavior Questionnaire-Revised.

Table S4. Associations between baseline clinical characteristics and study outcomes at enrollment.

Predictor	K-BDI-II	K-PSI-4 (Total)	K-PSI-4 (Parent)	K-PSI-4 (Child)	Self-Efficacy	IBQ-R (SUR)	IBQ-R (NEG)	IBQ-R (REG)
**Gestational age**	-0.05 (p=0.799; q=0.94)	-0.22 (p=0.233; q=0.69)	-0.07 (p=0.700; q=0.88)	-0.26 (p=0.145; q=0.64)	-0.21 (p=0.251; q=0.69)	+0.35 (p=0.037*; q=0.64)	+0.22 (p=0.206; q=0.69)	+0.15 (p=0.390; q=0.72)
**Birth weight**	+0.08 (p=0.635; q=0.84)	-0.16 (p=0.387; q=0.72)	-0.06 (p=0.725; q=0.90)	-0.18 (p=0.330; q=0.71)	-0.21 (p=0.248; q=0.69)	+0.20 (p=0.240; q=0.69)	+0.17 (p=0.325; q=0.71)	+0.16 (p=0.371; q=0.72)
**NICU stay**	+0.01 (p=0.949; q=0.97)	+0.09 (p=0.631; q=0.84)	-0.02 (p=0.909; q=0.96)	+0.17 (p=0.389; q=0.72)	+0.10 (p=0.596; q=0.81)	-0.27 (p=0.141; q=0.64)	-0.40 (p=0.023*; q=0.64)	-0.11 (p=0.546; q=0.77)
**Apgar 5-min**	-0.16 (p=0.414; q=0.72)	-0.32 (p=0.110; q=0.64)	-0.30 (p=0.124; q=0.64)	-0.31 (p=0.126; q=0.64)	+0.06 (p=0.772; q=0.92)	+0.14 (p=0.483; q=0.75)	-0.03 (p=0.875; q=0.96)	+0.08 (p=0.663; q=0.86)
**Infant female**	+0.29 (p=0.413; q=0.72)	+0.36 (p=0.314; q=0.71)	+0.48 (p=0.180; q=0.65)	+0.22 (p=0.532; q=0.77)	-0.62 (p=0.094; q=0.64)	+0.52 (p=0.141; q=0.64)	+0.58 (p=0.097; q=0.64)	+0.03 (p=0.934; q=0.96)
**Maternal age**	-0.33 (p=0.059; q=0.64)	-0.11 (p=0.549; q=0.77)	+0.02 (p=0.925; q=0.96)	-0.21 (p=0.260; q=0.69)	+0.06 (p=0.749; q=0.92)	-0.03 (p=0.875; q=0.96)	-0.29 (p=0.100; q=0.64)	+0.02 (p=0.908; q=0.96)
**Maternal education**	-0.26 (p=0.147; q=0.64)	-0.21 (p=0.250; q=0.69)	-0.20 (p=0.272; q=0.69)	-0.13 (p=0.502; q=0.75)	-0.19 (p=0.317; q=0.71)	-0.15 (p=0.411; q=0.72)	-0.13 (p=0.460; q=0.74)	-0.13 (p=0.454; q=0.74)
**Paternal education**	-0.28 (p=0.109; q=0.64)	-0.25 (p=0.168; q=0.65)	-0.39 (p=0.027*; q=0.64)	+0.04 (p=0.813; q=0.95)	+0.16 (p=0.392; q=0.72)	+0.13 (p=0.459; q=0.74)	-0.08 (p=0.676; q=0.87)	-0.14 (p=0.434; q=0.73)
**Household income**	-0.22 (p=0.212; q=0.69)	-0.17 (p=0.363; q=0.72)	-0.31 (p=0.088; q=0.64)	+0.04 (p=0.851; q=0.96)	+0.30 (p=0.109; q=0.64)	-0.02 (p=0.911; q=0.96)	-0.18 (p=0.327; q=0.71)	+0.14 (p=0.425; q=0.73)

Predictor	K-BDI-II	K-PSI-4 (Total)	K-PSI-4 (Parent)	K-PSI-4 (Child)	Self-Efficacy	IBQ-R (SUR)	IBQ-R (NEG)	IBQ-R (REG)
**Dual-income**	+0.29 (p=0.412; q=0.72)	+0.26 (p=0.468; q=0.74)	+0.01 (p=0.987; q=0.99)	+0.49 (p=0.187; q=0.65)	+0.41 (p=0.267; q=0.69)	-0.30 (p=0.389; q=0.72)	-0.02 (p=0.961; q=0.97)	-0.47 (p=0.187; q=0.65)
**Siblings present**	-0.14 (p=0.693; q=0.88)	+0.51 (p=0.158; q=0.65)	+0.22 (p=0.539; q=0.77)	+0.60 (p=0.098; q=0.64)	+0.07 (p=0.848; q=0.96)	-0.32 (p=0.354; q=0.72)	+0.65 (p=0.065; q=0.64)	-0.76 (p=0.031*; q=0.64)
**Maternal health**	-0.51 (p=0.242; q=0.69)	-0.82 (p=0.066; q=0.64)	-0.81 (p=0.068; q=0.64)	-0.46 (p=0.295; q=0.71)	+0.83 (p=0.078; q=0.64)	+0.67 (p=0.127; q=0.64)	-0.59 (p=0.178; q=0.65)	+0.25 (p=0.562; q=0.78)
**Primary day-to-day caregiver = parent**	+0.04 (p=0.913; q=0.96)	-0.04 (p=0.920; q=0.96)	-0.20 (p=0.590; q=0.81)	+0.08 (p=0.837; q=0.96)	+0.25 (p=0.503; q=0.75)	+0.11 (p=0.759; q=0.92)	+0.36 (p=0.327; q=0.71)	+0.25 (p=0.500; q=0.75)

Each cell reports the effect size, unadjusted p value, and FDR-adjusted q value. Continuous predictors are expressed as Pearson r and dichotomous predictors as Cohen's d. Benjamini–Hochberg FDR correction was applied across all 104 predictor–outcome tests. * Unadjusted $p < 0.05$. K-BDI-II: Korean Beck Depression Inventory-II; IBQ-R: Infant Behavior Questionnaire–Revised; K-PSI-4: Korean Parenting Stress Index, Fourth Edition; NICU: Neonatal intensive care unit.

Table S5. Associations between clinical characteristics and study outcomes at 12 months corrected age.

Predictor	K-BDI-II	K-PSI-4 (Total)	K-PSI-4 (Parent)	K-PSI-4 (Child)	Self-Efficacy	IBQ-R (SUR)	IBQ-R (NEG)	IBQ-R (REG)	BSID-II (MDI)	BSID-II (PDI)
**Gestational age**	-0.12 (p=0.490; q=0.90)	+0.02 (p=0.913; q=0.98)	+0.10 (p=0.591; q=0.94)	-0.11 (p=0.536; q=0.94)	-0.01 (p=0.954; q=0.98)	+0.36 (p=0.039*; q=0.42)	+0.05 (p=0.792; q=0.98)	+0.25 (p=0.154; q=0.67)	-0.02 (p=0.934; q=0.98)	+0.23 (p=0.261; q=0.77)
**Birth weight**	-0.05 (p=0.778; q=0.98)	-0.06 (p=0.728; q=0.98)	+0.02 (p=0.922; q=0.98)	-0.16 (p=0.388; q=0.83)	-0.08 (p=0.653; q=0.97)	+0.43 (p=0.013*; q=0.41)	+0.09 (p=0.609; q=0.95)	+0.38 (p=0.029*; q=0.41)	-0.12 (p=0.568; q=0.94)	+0.18 (p=0.388; q=0.83)
**NICU stay**	+0.07 (p=0.702; q=0.98)	-0.16 (p=0.405; q=0.83)	-0.20 (p=0.285; q=0.77)	+0.01 (p=0.962; q=0.98)	-0.17 (p=0.384; q=0.83)	-0.52 (p=0.003*; q=0.41)	-0.11 (p=0.577; q=0.94)	-0.22 (p=0.239; q=0.77)	-0.02 (p=0.929; q=0.98)	-0.12 (p=0.579; q=0.94)
**Apgar 5-min**	-0.27 (p=0.167; q=0.67)	-0.06 (p=0.748; q=0.98)	-0.09 (p=0.666; q=0.97)	-0.01 (p=0.959; q=0.98)	+0.05 (p=0.805; q=0.98)	+0.29 (p=0.144; q=0.67)	-0.13 (p=0.519; q=0.94)	+0.05 (p=0.811; q=0.98)	+0.12 (p=0.592; q=0.94)	+0.35 (p=0.117; q=0.59)
**Infant female**	+0.27 (p=0.454; q=0.87)	+0.80 (p=0.031*; q=0.41)	+0.65 (p=0.074; q=0.54)	+0.61 (p=0.094; q=0.58)	+0.01 (p=0.980; q=0.99)	-0.06 (p=0.862; q=0.98)	+0.65 (p=0.075; q=0.54)	-0.58 (p=0.111; q=0.58)	+0.39 (p=0.334; q=0.80)	+0.09 (p=0.812; q=0.98)
**Maternal age**	-0.43 (p=0.015*; q=0.41)	-0.32 (p=0.080; q=0.55)	-0.21 (p=0.256; q=0.77)	-0.36 (p=0.044*; q=0.44)	-0.25 (p=0.187; q=0.67)	+0.01 (p=0.964; q=0.98)	-0.35 (p=0.051; q=0.47)	+0.04 (p=0.836; q=0.98)	+0.07 (p=0.738; q=0.98)	+0.28 (p=0.170; q=0.67)
**Maternal education**	-0.46 (p=0.009*; q=0.41)	-0.37 (p=0.038*; q=0.42)	-0.39 (p=0.032*; q=0.41)	-0.24 (p=0.187; q=0.67)	+0.13 (p=0.490; q=0.90)	+0.19 (p=0.295; q=0.77)	-0.03 (p=0.892; q=0.98)	+0.15 (p=0.415; q=0.83)	-0.00 (p=0.996; q=1.00)	-0.06 (p=0.787; q=0.98)
**Paternal education**	-0.40 (p=0.026*; q=0.41)	-0.20 (p=0.282; q=0.77)	-0.29 (p=0.110; q=0.58)	-0.04 (p=0.841; q=0.98)	+0.34 (p=0.075; q=0.54)	+0.23 (p=0.205; q=0.72)	-0.08 (p=0.687; q=0.98)	+0.17 (p=0.366; q=0.83)	+0.18 (p=0.400; q=0.83)	+0.10 (p=0.644; q=0.97)
**Household income**	-0.30 (p=0.101; q=0.58)	-0.25 (p=0.174; q=0.67)	-0.39 (p=0.030*; q=0.41)	+0.05 (p=0.780; q=0.98)	+0.22 (p=0.256; q=0.77)	+0.14 (p=0.461; q=0.87)	-0.04 (p=0.814; q=0.98)	+0.19 (p=0.304; q=0.77)	-0.10 (p=0.626; q=0.97)	-0.08 (p=0.719; q=0.98)
**Dual-income**	+0.16 (p=0.667; q=0.97)	-0.14 (p=0.707; q=0.98)	-0.02 (p=0.945; q=0.98)	-0.32 (p=0.378; q=0.83)	+0.52 (p=0.172; q=0.67)	+0.20 (p=0.577; q=0.94)	+0.21 (p=0.562; q=0.94)	-0.16 (p=0.656; q=0.97)	+0.44 (p=0.288; q=0.77)	+0.43 (p=0.298; q=0.77)
**Siblings present**	-0.35 (p=0.323; q=0.79)	-0.08 (p=0.825; q=0.98)	+0.04 (p=0.912; q=0.98)	-0.32 (p=0.368; q=0.83)	+0.03 (p=0.927; q=0.98)	+0.52 (p=0.144; q=0.67)	+0.21 (p=0.556; q=0.94)	+0.04 (p=0.907; q=0.98)	-0.51 (p=0.211; q=0.72)	-0.41 (p=0.309; q=0.77)

Predictor	K-BDI-II	K-PSI-4 (Total)	K-PSI-4 (Parent)	K-PSI-4 (Child)	Self-Efficacy	IBQ-R (SUR)	IBQ-R (NEG)	IBQ-R (REG)	BSID-II (MDI)	BSID-II (PDI)
**Maternal health**	-0.38 (p=0.415; q=0.83)	-0.09 (p=0.845; q=0.98)	-0.17 (p=0.718; q=0.98)	+0.02 (p=0.962; q=0.98)	+0.07 (p=0.883; q=0.98)	+0.86 (p=0.069; q=0.54)	-0.47 (p=0.306; q=0.77)	+0.75 (p=0.112; q=0.58)	+0.47 (p=0.354; q=0.83)	+0.61 (p=0.232; q=0.77)
**Primary day-to-day caregiver = parent**	+0.30 (p=0.435; q=0.86)	+0.11 (p=0.780; q=0.98)	+0.05 (p=0.902; q=0.98)	+0.28 (p=0.455; q=0.87)	+0.04 (p=0.910; q=0.98)	-0.65 (p=0.092; q=0.58)	-0.21 (p=0.575; q=0.94)	-0.45 (p=0.244; q=0.77)	-0.97 (p=0.028*; q=0.41)	-0.62 (p=0.149; q=0.67)

Each cell reports the effect size, unadjusted p value, and FDR-adjusted q value. Continuous predictors are expressed as Pearson r and dichotomous predictors as Cohen's d. Benjamini–Hochberg FDR correction was applied across all 130 predictor–outcome tests. * Unadjusted $p < 0.05$. K-BDI-II: Korean Beck Depression Inventory-II; IBQ-R: Infant Behavior Questionnaire–Revised; K-PSI-4: Korean Parenting Stress Index, Fourth Edition; BSID-II: Bayley Scales of Infant Development, Second Edition; MDI: Mental Developmental Index; PDI: Psychomotor Development Index; NICU: Neonatal intensive care unit.

Sample sizes vary according to available predictor and outcome data; measure-level availability is summarized in Figure 1 of the main manuscript.

Table S6. Caregiver psychological, infant developmental and behavioral outcomes at 12 months according to neonatal brain injury category.

Outcome	None (n=20)	Grade1–2 GMH-IVH (n=7)	Grade 3–4 GMH-IVH/PVL (n=8)	p value	FDR q
**K-BDI-II**	14.4±8.6 (18)	12.3±5.1 (7)	12.9±4.8 (8)	0.751	0.808
**K-PSI-4 (Total)**	45.9±23.8 (18)	44.1±21.4 (7)	30.8±13.3 (8)	0.223	0.488
**K-PSI-4 (Parent)**	50.4±25.6 (18)	47.8±25.4 (7)	32.8±19.5 (8)	0.199	0.488
**K-PSI-4 (Child)**	40.4±23.3 (18)	39.8±16.2 (7)	33.6±14.1 (8)	0.808	0.808
**Self-efficacy**	3.1±0.2 (17)	3.0±0.1 (7)	3.0±0.2 (7)	0.767	0.808
**IBQ-R (SUR)**	4.6±0.7 (18)	4.6±0.7 (7)	4.8±0.5 (8)	0.775	0.808
**IBQ-R (NEG)**	3.5±0.5 (18)	3.9±0.5 (7)	3.6±0.7 (8)	0.244	0.488
**IBQ-R (REG)**	5.0±0.6 (18)	4.8±0.3 (7)	5.4±0.2 (8)	0.025*	0.250
**BSID-II (MDI)**	92.9±12.0 (16)	89.8±5.8 (4)	77.7±10.1 (6)	0.054	0.270
**BSID-II (PDI)**	84.1±14.0 (16)	87.0±3.5 (4)	76.3±15.4 (6)	0.376	0.627

Values are mean ± SD (number of participants with available data). Overall p-values were obtained using Kruskal–Wallis tests. Benjamini–Hochberg FDR correction was applied across the 10 subgroup outcome comparisons.

Table S7. Correlations among caregiver psychological well-being, infant temperament, and developmental outcomes at 12 months corrected age.

Variable 1	Variable 2	r/ρ	Squared coefficient (r^2 or $ρ^2$)	p	FDR q	n
**K-BDI-II**	K-PSI-4 (Total)	+0.55	0.30	<0.001*	0.007†	33
	K-PSI-4 (Parent)	+0.65	0.43	<0.001*	<0.001†	33
	K-PSI-4 (Child)	+0.16	0.02	0.386	0.600	33
	Self-Efficacy	+0.18	0.03	0.324	0.561	31
	IBQ-R (SUR)	-0.07	0.01	0.681	0.805	33
	IBQ-R (NEG)	+0.22	0.05	0.210	0.511	33
	IBQ-R (REG)	-0.24	0.06	0.170	0.449	33
	MDI	-0.11	0.01	0.607	0.772	26
	PDI	-0.30	0.09	0.142	0.426	26
**K-PSI-4 (Total)**	K-PSI-4 (Parent)	+0.92	0.85	<0.001*	<0.001†	33
	K-PSI-4 (Child)	+0.74	0.54	<0.001*	<0.001†	33
	Self-Efficacy	-0.03	0.00	0.879	0.899	31
	IBQ-R (SUR)	+0.06	0.00	0.730	0.805	33
	IBQ-R (NEG)	+0.47	0.22	0.006*	0.039†	33
	IBQ-R (REG)	-0.29	0.09	0.097	0.335	33
	MDI	+0.10	0.01	0.635	0.772	26
	PDI	-0.22	0.05	0.272	0.511	26
**K-PSI-4 (Parent)**	K-PSI-4 (Child)	+0.43	0.18	0.013*	0.073	33
	Self-Efficacy	-0.13	0.02	0.493	0.687	31
	IBQ-R (SUR)	+0.12	0.01	0.504	0.687	33
	IBQ-R (NEG)	+0.37	0.13	0.036*	0.160	33
	IBQ-R (REG)	-0.30	0.09	0.085	0.318	33
	MDI	+0.14	0.02	0.491	0.687	26
	PDI	-0.17	0.03	0.408	0.612	26

**K-PSI-4 (Child)**	Self-Efficacy	+0.17	0.03	0.363	0.596	31
	IBQ-R (SUR)	-0.10	0.01	0.572	0.756	33
	IBQ-R (NEG)	+0.42	0.18	0.015*	0.074	33
	IBQ-R (REG)	-0.20	0.04	0.261	0.511	33
	MDI	-0.04	0.00	0.844	0.883	26
	PDI	-0.24	0.06	0.235	0.511	26
**Self-Efficacy**	IBQ-R (SUR)	-0.20	0.04	0.286	0.515	31
	IBQ-R (NEG)	-0.25	0.06	0.168	0.449	31
	IBQ-R (REG)	-0.22	0.05	0.242	0.511	31
	MDI	-0.19	0.04	0.371	0.596	24
	PDI	-0.37	0.14	0.071	0.291	24
**IBQ-R (SUR)**	IBQ-R (NEG)	+0.21	0.04	0.244	0.511	33
	IBQ-R (REG)	+0.62	0.39	<0.001*	0.001†	33
	MDI	+0.06	0.00	0.771	0.826	26
	PDI	+0.23	0.05	0.254	0.511	26
**IBQ-R (NEG)**	IBQ-R (REG)	-0.09	0.01	0.634	0.772	33
	MDI	+0.07	0.00	0.733	0.805	26
	PDI	-0.02	0.00	0.918	0.918	26
**IBQ-R (REG)**	MDI	-0.08	0.01	0.711	0.805	26
	PDI	+0.31	0.10	0.120	0.387	26
**BSID-II (MDI)**	PDI	+0.61	0.38	<0.001*	0.007†	26

Pearson's correlation coefficient (r) or Spearman's rank correlation coefficient (ρ) was used, as appropriate. The square of the selected correlation coefficient (r^2 for Pearson or ρ^2 for Spearman) was calculated. Benjamini–Hochberg false discovery rate (FDR) correction was applied across the 45 selected correlation tests after the appropriate correlation method had been selected. * Unadjusted $p < 0.05$; † FDR-adjusted $q < 0.05$. K-BDI-II: Korean Beck Depression Inventory-II; IBQ-R: Infant Behavior Questionnaire–Revised; K-PSI-4: Korean Parenting Stress Index, Fourth Edition; BSID-II: Bayley Scales of Infant Development, Second Edition; MDI: Mental Developmental Index; PDI: Psychomotor Developmental Index.

Table S8. Significant cross-domain correlations among questionnaire subscales at 12 months.

Subscale 1	Subscale 2	Spearman ρ	ρ^2	p	FDR q	n
**K-PSI-4: Reinforces Parent (RE)**	IBQ-R: Approach	-0.65	0.42	<0.001*	0.0021†	33
	IBQ-R: Vocal Reactivity	-0.59	0.34	<0.001*	0.0075†	33
**K-PSI-4: Health (HE)**	IBQ-R: Activity Level	0.54	0.30	0.0011*	0.0180†	33
	IBQ-R: Distress to Limitations	0.53	0.28	0.0016*	0.0243†	33
	IBQ-R: Sadness	0.64	0.41	<0.001*	0.0026†	32

Only cross-domain K-PSI-4–IBQ-R correlations that remained significant after Benjamini–Hochberg FDR correction are presented. Each row reports Spearman's rank correlation coefficient (ρ), its squared coefficient (ρ^2), the unadjusted p value, and the FDR-adjusted q value. * Unadjusted $p < 0.05$; † FDR-adjusted $q < 0.05$.

Table S9. Longitudinal correlations between enrollment and 12-month outcomes.

Variable at enrollment	Variable at 12 months	r/ρ	Squared coefficient (r^2 or ρ^2)	p	FDR q	n
**K-BDI-II**	K-BDI-II	+0.55	0.30	0.001*	0.092	32
	K-PSI-4 (Total)	+0.23	0.05	0.209	0.462	32
	K-PSI-4 (Parent)	+0.27	0.07	0.134	0.413	32
	K-PSI-4 (Child)	+0.12	0.01	0.508	0.753	32
	Self-Efficacy	+0.13	0.02	0.503	0.753	30
	IBQ-R (SUR)	+0.14	0.02	0.438	0.677	32
	IBQ-R (NEG)	+0.22	0.05	0.235	0.494	32
	IBQ-R (REG)	-0.01	0.00	0.958	0.974	32
	BSID-II (MDI)	-0.09	0.01	0.653	0.791	26
	BSID-II (PDI)	-0.07	0.01	0.730	0.850	26
**K-PSI-4 (Total)**	K-BDI-II	+0.43	0.19	0.017*	0.245	30
	K-PSI-4 (Total)	+0.37	0.14	0.044*	0.311	30
	K-PSI-4 (Parent)	+0.39	0.16	0.031*	0.277	30
	K-PSI-4 (Child)	+0.20	0.04	0.284	0.568	30
	Self-Efficacy	-0.01	0.00	0.946	0.974	28
	IBQ-R (SUR)	+0.08	0.01	0.669	0.798	30
	IBQ-R (NEG)	+0.41	0.17	0.025*	0.245	30
	IBQ-R (REG)	-0.27	0.07	0.157	0.420	30
	BSID-II (MDI)	-0.26	0.07	0.195	0.456	26
	BSID-II (PDI)	-0.38	0.14	0.058	0.311	26
**K-PSI-4 (Parent)**	K-BDI-II	+0.49	0.24	0.006*	0.148	31
	K-PSI-4 (Total)	+0.41	0.16	0.024*	0.245	31
	K-PSI-4 (Parent)	+0.50	0.25	0.005*	0.148	31
	K-PSI-4 (Child)	+0.10	0.01	0.593	0.778	31
	Self-Efficacy	-0.11	0.01	0.561	0.774	29
	IBQ-R (SUR)	+0.04	0.00	0.824	0.903	31
	IBQ-R (NEG)	+0.26	0.07	0.163	0.420	31
	IBQ-R (REG)	-0.27	0.07	0.141	0.417	31

Variable at enrollment	Variable at 12 months	r/ρ	Squared coefficient (r² or ρ²)	p	FDR q	n
	BSID-II (MDI)	-0.07	0.00	0.738	0.850	26
	BSID-II (PDI)	-0.32	0.10	0.115	0.400	26
**K-PSI-4 (Child)**	K-BDI-II	+0.09	0.01	0.644	0.791	30
	K-PSI-4 (Total)	+0.23	0.05	0.214	0.462	30
	K-PSI-4 (Parent)	+0.11	0.01	0.561	0.774	30
	K-PSI-4 (Child)	+0.32	0.10	0.088	0.371	30
	Self-Efficacy	+0.00	0.00	0.993	0.993	28
	IBQ-R (SUR)	+0.11	0.01	0.576	0.778	30
	IBQ-R (NEG)	+0.47	0.22	0.008*	0.166	30
	IBQ-R (REG)	-0.11	0.01	0.551	0.774	30
	BSID-II (MDI)	-0.19	0.03	0.363	0.643	26
	BSID-II (PDI)	-0.37	0.14	0.062	0.311	26
Self-Efficacy	K-BDI-II	+0.21	0.04	0.261	0.536	30
	K-PSI-4 (Total)	-0.06	0.00	0.745	0.850	30
	K-PSI-4 (Parent)	-0.01	0.00	0.962	0.974	30
	K-PSI-4 (Child)	-0.18	0.03	0.350	0.643	30
	Self-Efficacy	+0.31	0.10	0.105	0.400	28
	IBQ-R (SUR)	-0.04	0.00	0.847	0.913	30
	IBQ-R (NEG)	-0.17	0.03	0.371	0.643	30
	IBQ-R (REG)	-0.05	0.00	0.792	0.880	30
	BSID-II (MDI)	-0.13	0.02	0.549	0.774	24
	BSID-II (PDI)	-0.30	0.09	0.161	0.420	24
**IBQ-R (SUR)**	K-BDI-II	-0.14	0.02	0.423	0.677	33
	K-PSI-4 (Total)	+0.14	0.02	0.437	0.677	33
	K-PSI-4 (Parent)	+0.06	0.00	0.754	0.850	33
	K-PSI-4 (Child)	+0.23	0.05	0.200	0.456	33
	Self-Efficacy	+0.25	0.06	0.175	0.436	31
	IBQ-R (SUR)	+0.40	0.16	0.020*	0.245	33
	IBQ-R (NEG)	-0.14	0.02	0.440	0.677	33
	IBQ-R (REG)	+0.30	0.09	0.088	0.371	33

Variable at enrollment	Variable at 12 months	r/ρ	Squared coefficient (r² or ρ²)	p	FDR q	n
	BSID-II (MDI)	+0.11	0.01	0.587	0.778	26
	BSID-II (PDI)	+0.30	0.09	0.133	0.413	26
**IBQ-R (NEG)**	K-BDI-II	+0.18	0.03	0.321	0.611	33
	K-PSI-4 (Total)	+0.34	0.11	0.056	0.311	33
	K-PSI-4 (Parent)	+0.34	0.11	0.054	0.311	33
	K-PSI-4 (Child)	+0.27	0.07	0.126	0.413	33
	Self-Efficacy	+0.09	0.01	0.641	0.791	31
	IBQ-R (SUR)	+0.16	0.03	0.366	0.643	33
	IBQ-R (NEG)	+0.33	0.11	0.060	0.311	33
	IBQ-R (REG)	-0.16	0.03	0.378	0.643	33
	BSID-II (MDI)	-0.32	0.10	0.111	0.400	26
	BSID-II (PDI)	-0.36	0.13	0.068	0.319	26
**IBQ-R (REG)**	K-BDI-II	+0.19	0.03	0.299	0.584	33
	K-PSI-4 (Total)	+0.01	0.00	0.936	0.974	33
	K-PSI-4 (Parent)	-0.03	0.00	0.856	0.913	33
	K-PSI-4 (Child)	+0.08	0.01	0.651	0.791	33
	Self-Efficacy	+0.24	0.06	0.192	0.456	31
	IBQ-R (SUR)	+0.15	0.02	0.412	0.677	33
	IBQ-R (NEG)	-0.29	0.08	0.102	0.400	33
	IBQ-R (REG)	+0.35	0.12	0.047*	0.311	33
	BSID-II (MDI)	-0.29	0.08	0.150	0.420	26
	BSID-II (PDI)	-0.10	0.01	0.617	0.791	26

Pearson's correlation coefficient (r) or Spearman's rank correlation coefficient (ρ) was used, as appropriate. The square of the selected correlation coefficient (r^2 for Pearson or ρ^2 for Spearman) was calculated. Benjamini–Hochberg false discovery rate (FDR) correction was applied across the 80 longitudinal correlation tests after the appropriate correlation method had been selected. * Unadjusted $p < 0.05$; † FDR-adjusted $q < 0.05$. K-BDI-II: Korean Beck Depression Inventory-II; IBQ-R: Infant Behavior Questionnaire–Revised; K-PSI-4: Korean Parenting Stress Index, Fourth Edition; BSID-II: Bayley Scales of Infant Development, Second Edition; MDI: Mental Developmental Index; PDI: Psychomotor Developmental Index.
